# Mixed Endometrial Epithelial Carcinoma: Epidemiology, Treatment and Survival Rates—A 10-Year Retrospective Cohort Study from a Single Institution

**DOI:** 10.3390/jcm12196373

**Published:** 2023-10-05

**Authors:** Christina Pappa, Valentina Le Thanh, Sarah Louise Smyth, Andreas Zouridis, Ammara Kashif, Negin Sadeghi, Alisha Sattar, Stephen Damato, Mostafa Abdalla, Antonio Simone Laganà, Federico Ferrari, Sean Kehoe, Susan Addley, Hooman Soleymani majd

**Affiliations:** 1 Department of Gynaecological Oncology, Oxford University Hospitals NHS Foundation Trust, Oxford OX3 7LE, UK; christina.pappa@ouh.nhs.uk (C.P.); valentina.lethanh@ouh.nhs.uk (V.L.T.); andreas.zouridis@ouh.nhs.uk (A.Z.); ammara.kashif@ouh.nhs.uk (A.K.); sean.kehoe@ouh.nhs.uk (S.K.); 2 Gynaecology—Guy’s and St Thomas’ NHS Foundation Trust, London SE1 9RT, UK; mostafa.abdalla@nhs.net; 3 Unit of Obstetrics and Gynecology, “Paolo Giaccone” Hospital, Department of Health Promotion, Mother and Child Care, Internal Medicine and Medical Specialties (PROMISE), University of Palermo, 90127 Palermo, Italy; antoniosimone.lagana@unipa.it; 4 Department of Clinical and Experimental Sciences, University of Brescia, 25136 Brescia, Italy; federico.ferrari@unibs.it; 5 Department of Gynaecological Oncology, University Hospitals of Derby and Burton NHS Foundation Trust, Derby DE22 3NE, UK; susan.addley@nhs.net

**Keywords:** mixed endometrial carcinoma, high grade, recurrence, clear cell, serous, survival

## Abstract

Mixed endometrial carcinoma (MEEC) refers to rare endometrial tumours that are composed of two or more distinct histotypes, at least one of which is serous or clear cell. The aim of this study was to evaluate the epidemiology, treatment outcomes and survival rates of patients with mixed endometrial carcinoma. The medical records of 34 patients diagnosed with MEEC between March 2010 and January 2020 were reviewed retrospectively. Clinicopathological variables and treatment strategies were assessed, and overall survival and disease-free survival rates were evaluated. The histology of endometrioid and serous component was found in 26 (76.5%) patients, followed by serous and clear-cell components (5/34, 14.5%) and mixed endometrioid serous and clear-cell components (3/34, 8.8%). The median age at diagnosis was 70 years (range 52–84), and the median follow-up time was 55 months. The 5-year disease-free survival and the 5-year overall survival were 50.4% and 52.4%, respectively. Advanced disease stage was identified as an independent predictor of inferior disease-free (<0.003) and overall survival (*p* < 0.001). Except for stage, none of the traditional prognostic factors was associated with disease recurrence or death from disease. MEECs represent rare high-risk endometrial carcinomas with significant diagnostic and treatment challenges. Undoubtedly, the implementation of a molecular analysis can offer further diagnostic and management insights.

## 1. Introduction

Endometrial cancer represents the most common gynaecological malignancy in the developed world, with a globally increased incidence due to the extended life expectancy and possibly the high prevalence of associated risk factors [1]. It is the sixth most diagnosed cancer in women, with 417,000 new cases and 97,000 deaths in 2020 [2].

Mixed epithelial endometrial carcinoma (MEEC) is a rare entity, accounting for less than 3% of the epithelial endometrial malignancies. The 2020 World Health Organization (WHO) classification defines mixed endometrial carcinoma as a tumour composed of two or more spatially distinct histological types, at least one of which is serous or clear-cell carcinoma [3]. The two components should be unambiguously morphologically and immunohistochemically distinct histotypes of endometrial carcinoma [4]. Mixed endometrial carcinomas are regarded as high-grade neoplasms by definition, and they exhibit aggressive behaviour, resulting in a poor prognosis [5,6].

The proportion of each cell type can vary widely between tumours, and this can impact the prognosis and treatment of the cancer. The diagnosis and management of MEEC can be challenging, as it is difficult to distinguish between the different types of epithelial cells and determine the best treatment approach. The histological classification following the morphological characterization of the tumour, allied with confirmatory immunohistochemistry, particularly among the high-grade histotypes, is frequently hampered by overlap between histologic subtypes and significant interobserver variability and poor reproducibility even among the most experienced pathologists [6,7,8].

Treatment for MEEC typically involves surgical removal of the tumour, along with adjuvant chemotherapy or radiotherapy, depending on the stage and characteristics of the cancer [5,9,10]. Prognosis can be difficult to predict in MEEC due to the varied cell types and molecular features, but certain factors, such as the presence of clear-cell carcinoma or lymph node involvement, have been associated with poorer prognosis [11,12].

The rationale for accurately diagnosing mixed endometrial carcinoma is that even a minor component of serous or clear-cell carcinoma within an otherwise typical endometrioid carcinoma may confer an adverse outcome similar to a pure serous or clear-cell carcinoma [13,14,15].

There are limited data available specifically on the recurrence rates for mixed epithelial endometrial carcinoma (MEEC), as it is a rare and relatively understudied subtype of endometrial cancer [16,17].

The aim of the study was to assess and present the 10-year experience of the epidemiology, treatment outcomes and survival patterns of patients with mixed epithelial endometrial carcinoma managed in a single tertiary cancer referral centre in the United Kingdom.

## 2. Materials and Methods

This study represents a retrospective analysis of women with mixed epithelial endometrial carcinoma treated in Oxford University Hospitals, NHS Foundation Trust, a tertiary cancer referral centre, between March 2010 and January 2020.

The study was registered as a service evaluation project (registration number 6604) with no patient identifiable data. Informed written consent for potential future research purposes was obtained from all the participants at the time of the operation. The service evaluation protocol was registered in accordance with the Oxford University Hospitals Trust requirements. The design, analysis, interpretation of data, drafting and revisions conform to the Helsinki Declaration, the Committee on Publication Ethics’ guidelines (http://publicationethics.org/, accessed on 10 March 2023) and the Reporting of Studies Conducted Using Observational Routinely Collected Health Data (RECORD) Statement validated by the Enhancing the Quality and Transparency of Health Research Network (www.equator-network.org, accessed on 10 March 2023) [18].

Women with a histological diagnosis of mixed epithelial endometrial carcinoma and no previous surgery concerning endometrial malignancy (primary surgery) were recruited. Patients with histology other than mixed epithelial endometrial carcinoma and patients with inadequate follow-up data were excluded.

We retrospectively reviewed and analysed the medical records of the patients diagnosed with mixed endometrial carcinoma. The initial diagnosis was made through targeted hysteroscopic biopsy, pipelle endometrial biopsy or endometrial curettage, obtaining as much material as possible, according to our previously published experience [19]. Subsequently, all patients had imaging with magnetic resonance imaging (MRI) and/or a computerized tomography (CT) scan to assess locoregional or systemic spread of the disease. The treatment plan was implemented following the review of the multidisciplinary team (MDT).

All eligible patients had surgical management with total hysterectomy and bilateral salpingo-oophorectomy. Full surgical staging, including pelvic and para-aortic lymphadenectomy and omentectomy plus/minus peritoneal washing, was performed based on clinical judgement, depending on the patient’s performance status.

The extracted data included the age; body mass index (BMI); underlying comorbidities of the patient; length of hospitalization; and information about the surgical procedure, such as the type of surgery, surgical time and estimated blood loss. With regard to the tumour, we recorded the initial diagnosis; histological type; tumour dimensions; presence of lymphovascular space invasion (LVSI); presence of cervical, adnexal or parametrial invasion; status and number of lymph nodes; status of omentum and cytology; and the final staging. Intraoperative, acute and late postoperative complications were also recorded. For the patients who received adjuvant therapy, we recorded the type of therapy, the regiment and the associated complications, whenever available.

Follow-up was recorded in months from the date of surgery up to the study period when the patients were last tracked. Follow-up was initially every 3 months for the first 2 years; every 6 months from year 3 to year 5; and then annually, if required. Each clinical visit included history taking, investigation for signs and symptoms suspicious of disease relapse and clinical examination for the detection of treatment complications and assessment of evidence of recurrence.

Disease-free survival (DFS) was measured from the date of surgery until the date of first recurrence or death from any cause, and overall survival (OS) was measured from the diagnosis date until death.

We performed a statistical analysis using the IBM SPSS Statistics 29.0.1.0 version software. Continuous variables are presented as mean ± standard deviation or median (range), and categorical variables are presented using frequency (n) and percentage (%). Descriptive statistics were used to summarize the demographic and clinical characteristics of patients. An independent *t*-test was used to compare the continuous variable. Furthermore, classified variables were analysed using the chi-square (χ2) test. The Kaplan–Meier method was used to perform disease-free survival (DFS) and overall survival (OS) analyses, and log-rank tests were used to compare survival rates. Potential risk factors for recurrence and mortality were analysed and assessed using univariate and multivariate Cox regression analyses. A *p*-value < 0.05 was considered to be statistically significant.

## 3. Results

A total of 863 patients were surgically managed for endometrial carcinoma in our centre between March 2010 and January 2020. There were 35 women who were diagnosed with mixed epithelial endometrial carcinoma, representing less than 4% of the uterine malignancies treated in our unit, during the study period. Among them, one was excluded due to missing data.

Therefore, a total of 34 patients were included in the study. The basic characteristics of the patients and the histopathological and treatment characteristics are presented in Table 1.

The mean age at diagnosis was 70 years (range 58–84), and 44.1% of the patients had a BMI over 30 kg/m^2^. All of the women were postmenopausal, 61% of them had known hypertension (HTN) and 14% also had a diagnosis of breast cancer with unknown BRCA status. Underlying diabetes was recorded in 2 patients and 4 patients were recorded as smokers. Average time interval from the first MDT meeting to the day of the surgery was 55 days.

Laparoscopy was performed in 27 patients. Seven patients (20.6%) underwent laparotomy, three of whom after intraoperative conversion due to multiple previous abdominal surgeries and extended adhesions. Twenty-seven patients had pelvic lymphadenectomy, and two underwent para-aortic lymphadenectomy. Seven patients declined lymphadenectomy. On average, 13 pelvic lymph nodes were harvested (range 7–35). Lymph node histology was positive for disease invasion in seven patients (20.6%), and 70% of patients (24/34) also had omentectomy.

The mean length of hospitalization was 4 days (range 1–11 days), and the mean estimated blood loss was 150 mL.

The most common histopathology was represented by endometrioid and serous components (26/34, 76.5%), followed by serous and clear-cell components (5/354 14.5%) and a mixture of endometrioid, serous and clear-cell components (3/34, 8.8%). All of the endometrioid components were high-grade tumours. Twenty-eight patients (82.4%) received adjuvant treatment following surgical management; eight had vault brachytherapy (VBT), sixteen received a combination of platinum- and taxane-based chemotherapy (CT) and radiotherapy (EBRT) and four received external beam radiotherapy (EBRT) only. Adjuvant treatment was declined in six cases.

The median follow-up was 55 months (range 1–144). Twelve recurrences (35.2%) were recorded, with mean time of recurrence being 11 (range 2–40) months after the completion of the initial treatment. Single vault recurrence was reported in one patient, three women developed lung recurrence and eight women had nodal pelvic and/or extrapelvic recurrence. Eighteen deaths were recorded (52.9%), with 82.4% of the patients having died within 15 months after the initial treatment. The characteristics of the recurrent cases are presented in Table 2.

The univariate Cox regression analysis indicated that the risk of recurrence and the risk of death from disease are both correlated with an advanced stage of disease (HR = 5.05, 95% CI 1.80–14.30, and *p* = 0.022; and HR = 3.29, 95% CI 1.42–9.79, and *p* = 0.032, respectively). The univariate Cox proportional hazards analysis for the risk of recurrence and disease-specific death is presented in Table 3.

The multivariate Cox regression analysis after the adjustment of the covariates for age, associated comorbidities, surgical approach, pelvic lymph node dissection, number of retrieved lymph nodes, administration of adjuvant treatment, depth of myometrial invasion, adnexal involvement, breaching of the uterine serosa, parametrial involvement, pelvic lymph node involvement, para-aortic lymph node involvement and lymphovascular space invasion (LVSI) confirmed that the stage of the disease is independently related to the risk of recurrence (HR = 12.86; 95% CI 2.76–217.67; *p* < 0.003) and the risk of death from disease (HR = 15.57; 95% CI 3.16–246.50; *p* < 0.001).

**Figure 1:**
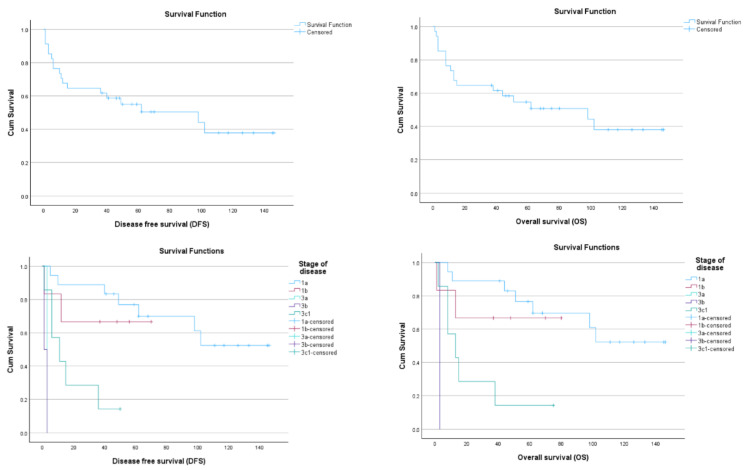
Kaplan–Meier curves for disease-free survival (DFS) (left) and overall survival (OS) (right) for the population study (above) and for the five different stages (below).

The 5-year disease-free survival rate (DFS) in our study was 50.4% (95% CI 34.76–6.67), and the 5-year overall survival (OS) rate was 52.4% (95% CI 36.92–78.43) (Figure 1).

We classified the complications as intraoperative, acute and late postoperative. There were two cases with intraoperative diathermy injury to the obturator nerve. Acute postoperative complications recorded in three cases, including one case of grade IV complication (ischemic stroke) and two cases of grade II complication (cellulitis and haematoma), as classified by the Clavien–Dindo classification of surgical complications [20]. Regarding late postoperative complications, three patients (8.8%) developed lymphatic-associated complications in the form of lymphoedema.

## 4. Discussion

Mixed epithelial endometrial carcinoma (MEEC) is a rare and challenging subtype of endometrial cancer that requires careful diagnosis and treatment planning to achieve the best possible outcomes for patients.

To the best of our knowledge, there are limited data available that focus on the recurrence and survival rates of mixed epithelial endometrial carcinoma. However, studies have reported on the overall recurrence rates for high-grade endometrial cancer cases, which can include groups of MEEC. In our study, the overall 5-year survival rate was 52.4%, which is consistent with the literature regarding non-endometrioid endometrial carcinomas [16,21,22]. Li et al. retrospectively studied 890 patients with stage IA endometrial carcinoma, including 47 patients with mixed endometrial pathology. Compared with the endometrioid subtype, mixed endometrioid and non-endometrioid subtypes had inferior survival outcomes [13].

One recent study analysed the recurrence patterns and risk factors for 834 patients with endometrial cancer, including 48 patients with mixed histology tumours. The study found that the 5-year recurrence rate for all patients with endometrial cancer was 14.1%. For patients with mixed histology tumours, the 5-year recurrence rate was 33.3%. The researchers also identified several risk factors for recurrence, including advanced stage, lymph node involvement and high-grade tumours [6].

In our study, we found that the disease-free survival (DFS) and the overall survival (OS) are independently related to the stage of the disease (*p* < 0.003 and < 0.001, respectively), with the 5-year overall survival being 66.7% vs. 14.3% for stage I and stage III disease, respectively. The 5-year disease-free survival (DFS) was found to be 69.9% in stage I disease and 14.3%in stage III disease.

Following the univariate and multivariate Cox regression analyses performed on our sample, except for stage (*p* < 0.001), none of the traditional prognosticators was independently associated with disease recurrence or death from disease. This could most possibly be explained by the small sample of mixed endometrial cases in our cohort.

Most patients diagnosed with mixed endometrial carcinoma can be treated with a laparoscopic approach, with less complication rates and a shorter hospital stay. In our study, we found that both laparoscopic and open approaches have equivalent oncologic outcomes, a result that is consistent with the current literature. Several studies comparing laparoscopy and laparotomy for early-stage endometrial cancer have shown non-inferior oncological outcomes with decreased estimated blood loss, less pain, shorter hospitalization and enhanced recovery following minimal invasive surgery when compared with open procedures [23,24]. The data that are available comparing minimally invasive and open surgery specifically in high-risk and advanced-stage endometrial cancer cases have shown no significant difference in the associated peri- and post-operative morbidity, as well as in the oncologic outcomes, between the two routes of surgery [25,26]. However, based on well-based evidence, uterine manipulator should be avoided in minimally invasive techniques, as it has been proven to be associated with worse oncologic outcomes. [27,28]

In our cohort, we found no significance of systematic lymphadenectomy in the overall and recurrence-free survival rates. Retrospective studies have shown a survival benefit following systematic pelvic and para-aortic lymphadenectomy in patients with high-risk-features endometrial carcinoma [29,30]. However, those results might bear a high risk of bias as the therapeutic and prognostic value of systematic lymphadenectomy in high-risk endometrial cancer has not been investigated in prospective randomized trials.

In the future, we hope that the completion of the open-label, randomized, controlled trial Endometrial Cancer Lymphadenectomy Trial (ECLIAT) will help us to ascertain whether systematic pelvic and para-aortic lymphadenectomy has a significant impact on overall survival (OS) in patients with a high risk of recurrence of endometrial cancer FIGO stages I and II [31].

Following the recent revision of the endometrial cancer staging system, we attempted to reallocate the disease stage according to the new staging system, FIGO 2023, to assess the potential stage migration and potential impact it might have on the recommended treatment pathway [32]. Mixed epithelial endometrial carcinomas are considered, by definition, to be aggressive histological types and therefore would have been staged as at least IC according to the new staging system. In our study, all of the cases that were staged as stage IA (18/34) and IB (6/34) involved at least some extent of myometrial invasion, which classifies them as stage IIC FIGO stage 2023 (24/34), as they represent aggressive histological types with any myometrial involvement. There was one case with proven adnexal involvement which was staged as IIIA and would currently be staged as IIIA1 disease based on the FIGO 2023 staging system. Stage IIIB disease was found in two cases, and both would be classified as stage IIIb1 according to the new 2023 staging system, due to vaginal and parametrial involvement. Finally, all seven cases with macro-metastasis in the pelvic lymph nodes (stage IIIC as per FIGO 2009) would currently represent stage IIIC1ii disease based on the FIGO 2023 staging system. Stage upgrading to stage IIC according to the FIGO 2023 staging system occurred in all early-stage cases with IA and IB FIGO 2009 disease (24/34, 70.6%), while stages IIIA (1/34, 2.9%), IIIB (2/34,5.9%) and IIIC (7/34, 20.6%) disease, according to FIGO 2009 staging system, either remained unchanged or were classified under specific newly added subcategories, according to the new staging system. Unfortunately, data regarding molecular classification and characteristics were very limited in our study, and they could not be safely integrated into the staging process to reflect their value in the treatment recommendation.

During the last decade, the stratification of endometrial cancer has moved forward and leaped from the histopathological classification based on tumour morphology and tumour grade to a genomic-based classification, as described by The Cancer Genome Atlas (TCGA) in 2013. Four distinct prognostic endometrial cancer groups were defined, namely POLE ultra-mutated, MSI (microsatellite instability) hyper-mutated, copy-number low and copy-number high, based on a combination of whole-genome or exome sequencing, microsatellite instability (MSI) assays and copy number analysis. [33]

One step ahead, with the view to create a more cost-effective and easily applicable method in more diagnostic settings, the ProMisE (Proactive Molecular Risk Classifier for Endometrial Cancer) and the TransPORTEC initiative have developed and validated a stratification system that can be applied to diagnostic specimens. Using a combination of immunohistochemistry for mismatch repair (MMR), focused next-generation sequencing (NGS) for detection of pathogenic POLE and immunohistochemistry for the p53 protein in a common diagnostic specimen, patients are categorized according to one of four distinct prognostic subtypes, which further direct adjuvant therapy and potential prognosis [34,35,36].

The molecular classification of the PORTEC-3 trial population has confirmed the strong prognostic value and the benefit from combined adjuvant therapy in p53abnormal tumours, regardless of the histologic type. Furthermore, it has shown that POLE-ultramutated (POLEmut) tumours had excellent outcomes, with almost no recurrence in both arms regarding the 5-year recurrence-free survival, following combined adjuvant chemotherapy and radiotherapy (CTRT) versus radiotherapy alone (RT), being 100% versus 97% [37]. The identification of mismatch repair deficiencies (dMMR) or high microsatellite instability (MSI-H) is found in a subset of endometrial cancers and is associated with a higher response rate to immune checkpoint inhibitors, such as pembrolizumab [38,39]. Furthermore, in absence of a specific molecular profile, the identification of L1CAM can be a useful predictor of early relapse in high risk endometrial cancer type [40], and, similarly, the use of machine learning algorithms, using multiple classical parameters, can help to build a prognostic model [41].

Overall, the retrospective nature and the small sample size are the main limitations of our study. The results should be interpreted with caution, as the absence of statistical significance regarding the role of the different risk factors and variables in the recurrence and survival rates might be attributed to the small sample size. To our knowledge, this is the first study evaluating data exclusively for mixed epithelial endometrial carcinoma.

The management of mixed epithelial endometrial carcinoma (MEEC) presents several challenges for clinicians and researchers that need to be addressed to improve oncologic outcomes for patients. Accurate and early diagnosis plays a fundamental role in determining the appropriate treatment plan. Improved diagnostic techniques, such as molecular profiling and imaging technologies, can help clinicians to effectively stratify the patients and tailor subsequent treatment [42,43]. The heterogeneity of MEEC justifies individualized treatment plans that consider the specific characteristics and biological behaviour of the tumour. This may involve a combination of surgery, chemotherapy and radiation therapy, as well as targeted therapies based on the molecular profile of the tumour. [44] The identification of specific biomarkers associated with the distinct features of mixed endometrial carcinoma can guide treatment decisions and predict outcomes for patients [45,46]. Research is ongoing to identify potential biomarkers, such as mutations in certain genes or expression of specific proteins, that may be useful in the diagnosis and treatment of MEEC [47]. The need for more clinical trials that are specifically focused on MEEC to evaluate the effectiveness of different treatment approaches and identify new therapies is undeniable, and collaboration between researchers and clinicians is essential to design and implement clinical trials that address the unique challenges of MEEC. Finally, patient education and support, including clinicians and patient advocacy groups, will be invaluable to help patients to navigate their treatment options and manage the emotional and psychological impact of a rare and complex cancer diagnosis.

The global vision of the Gynaecological Oncology Committee and the allied specialties should be to go beyond the tumour phenotype and decode and consolidate the tumour biology to develop prognostic algorithms and individualized treatment approaches, with the aim of improving the oncologic outcomes and the quality of life of the patients and their families.

## Figures and Tables

**Table 1:** Demographic data, treatment details and clinicopathological characteristics.

		N (%)	Recurrence (N of each Subgroup)	*p*-Value	Overall Mortality (N of each Subgroup)	*p*-Value
**Demographic data**	**Age**			0.301		0.288
	<65	6 (17.6)	5		3	
	≥65	28 (82.4)	7		15	
	**BMI**			0.222		0.203
	<30	19 (55.9)	6		10	
	≥30	15 (44.1)	6		8	
	**Associated comorbidities**					
	HTN	4 (11.8)	1	0.854	2	0.801
	DM	2 (5.9)	0	0.908	0	0.902
	COPD	4 (11.8)	2	0.885	2	0.813
	Breast cancer	5 (14.7)	3	0.113	2	0.071
	Smoke	4 (11.8)	2	0.801	3	0.768
**Treatment details**	**Surgical approach**			0.325		0.346
	Laparoscopy	27 (79.4)	9		13	
	Laparotomy	7 (20.6)	3		5	
	**Pelvic lymph node dissection**			0.203		0.192
	No	7 (20.6)	3		5	
	Yes	27 (79.4)	9		13	
	**Adjuvant treatment**			0.209		0.103
	No	6 (17.6)	1		4	
	Yes	28 (82.4)	11		14	
**Histological features**	**Histological type**					
	E + S	26 (76.5)	8	0.789	12	0.586
	S + CC	5 (14.7)	3		4	
	E + S + CC	3 (8.8)	1		2	
	**FIGO stage**			**<0.003**		**<0.001**
	IA	18 (53)	2		7	
	ΙΒ	6 (17.6)	1		2	
	ΙΙΙA	1(2.9)	1		1	
	ΙΙΙΒ	2 (5.9)	2		2	
	ΙΙΙC1	7 (20.6)	6		6	
	**FIGO stage category**			**<0.021**		**<0.011**
	Early (I)	24 (70.6)	3		9	
	Advanced (II–IV)	10 (29.4)	9		9	
	**Depth of myometrial invasion**			0.834		0.775
	<50%	23 (67.6)	5		10	
	≥50%	11 (32.4)	7		8	
	**Cervical stroma involvement**			0.141		0.987
	No	30 (88.2)	9		15	
	Yes	4 (11.8)	3		3	
	**Adnexal involvement**			0.178		0.137
	No	33 (97.1)	11		17	
	Yes	1 (2.9)	1		1	
	**Serosal breach**			0.500		0.514
	No	30 (88.2)	8		14	
	Yes	4 (11.8)	4		4	
	**Parametrial involvement**			0.101		0.111
	No	33 (97.1)	11		17	
	Yes	1 (2.9)	1		1	
	**Pelvic lymph node involvement**			0.482		0.503
	No	20 (58.8)	5		11	
	Yes	7 (20.6)	6		6	
	Unknown	7 (20.6)	1		1	
	**Paraaortic lymph node involvement**			0.482		0.588
	No	2 (5.9)	0		0	
	Yes	0 (0)	0		0	
	Unknown	32 (94.1)	12		18	
	**LVSI**			0.063		0.057
	No	15 (44.1)	3		6	
	Yes	19 (55.9)	9		12	

S, serous endometrial carcinoma; E, endometrioid endometrial carcinoma; CC, clear-cell endometrial carcinoma; LVSI, lymphovascular space invasion; HTN, hypertension; DM, diabetes mellitus; COPD, chronic obstructive pulmonary disease. Statistically significant results are highlighted in bold (*p*-value < 0.05).

**Table 2:** Characteristics of recurrent cases of mixed epithelial endometrial carcinoma.

No.	Histology	Stage	Myometrial Invasion	Cervical Involvement	Adnexal Involvement	Lymph Node Involvement	LVSI	VBT	EBRT	CT	Site of Recurrence	DFS	OS
1	S + CC	1B	Yes	No	No	No	No	Yes	Yes	No	Pelvic LN Peritoneal deposits	12	13
2	E + S	1A	No	No	No	No	Yes	No	No	No	Single Vault Recurrence	40	44
3	E + S	3C1	Yes	No	No	Yes	Yes	No	Yes	Yes	Extrapelvic LN Peritoneal deposits	6	8
4	E + S	1A	No	No	No	No	Yes	Yes	No	No	Pelvic LN	10	11
5	E + S + CC	3A	Yes	No	Yes	No	Yes	No	No	No	Extrapelvic LN Lung Peritoneal deposits	3	3
6	E + S	3C1	Yes	Yes	No	Yes	Yes	No	Yes	Yes	Extrapelvic LN	6	8
7	S + CC	3B	Yes	Yes	No	No	Yes	No	No	No	Lung	1	3
8	S + CC	3B	Yes	Yes	No	UK	Yes	No	No	No	Extrapelvic LN	3	3
9	E + S	3C1	No	No	No	Yes	No	No	Yes	Yes	Pelvic LN	15	15
10	E + S	3C1	No	No	No	Yes	No	No	No	No	Lung	1	2
11	E + S	3C1	Yes	No	No	Yes	Yes	No	Yes	Yes	Pelvic LN	36	38
12	E + S	3C1	No	No	No	Yes	Yes	Yes	Yes	Yes	Extrapelvic LN	11	13

S, serous endometrial carcinoma; E, endometrioid endometrial carcinoma; CC:, clear-cell endometrial carcinoma; UK, unknown; LVSI, lymphovascular space invasion; VBT, vaginal vault brachytherapy; EBRT, external beam radiotherapy; CT, chemotherapy; LN, lymph nodes; DFS, disease-free survival; OS, overall survival.

**Table 3:** Univariate Cox proportional hazards analysis for the risk of recurrence and disease-specific death for mixed epithelial endometrial cancer cases.

	Recurrence		Death from Disease	
	HR (95% CI)	*p*-Value	HR (95% CI)	*p*-Value
**AGE**	1.02 (0.99–1.06)	0.204	1.02 (0.99–1.05)	0.310
**BMI**	1.05 (0.88–1.25)	0.614	1.11 (0.96–1.29)	0.170
**Associated comorbidities**				
HTN	1.04 (0.97–1.12)	0.291	1.02 (0.96–1.08)	0.605
DM	1.01 (0.98–1.02)	0.798	1.02 (1.01–1.03)	0.216
COPD	1.96 (0.44–8.42)	0.378	5.39 (2.28–11.96)	0.101
Breast cancer	2.20 (1.05–8.57)	0.106	2.08 (1.11–10.89)	0.223
Smoke	1.85 (0.34–3.01)	0.646	0.85 (0.78–2.11)	0.104
**Surgical approach**				
Laparoscopy				
Laparotomy	1.04 (0.97–1.12)	0.291	1.02 (0.96–1.08)	0.605
**Pelvic lymph node dissection**				
No				
Yes	0.85 (0.33–2.02)	0.667	0.57 (0.31–1.22)	0.094
**Number of LN removed**	0.97 (0.93–1.01)	0.149	0.96 (0.93–0.99)	0.142
**Adjuvant treatment**				
No				
Yes	1.39 (0.53–3.68)	0.483	0.89 (0.41–1.85)	0.739
**Histological type**				
E + S	2.93 (0.98–10.79)	0.059	3.78 (3.87–12.06)	0.107
S + CC	3.11 (1.02–12.13)	0.057	3.648 (1.22–10.89)	0.123
E + S + CC	3.62 (1.27–10.34)	0.066	2.33 (0.88–6.15)	0.088
**FIGO stage**				
IA				
ΙB	3.00 (0.95–9.50)	0.062	2.40 (0.87–6.65)	0.091
ΙΙΙA	4.93 (0.98–24.79)	0.053	10.78 (3.87–30.06)	**<0.001**
ΙΙΙB	9.38 (1.08–81.33)	**0.042**	9.06 (1.91–42.98)	**0.006**
IIIC1	15.36 (3.00–78.92)	**0.001**	7.10 (1.48–34.00)	**0.014**
**FIGO stage category**				
Early (I)				
Advanced (II-IV)	5.05 (1.80–14.30)	**0.022**	3.29 (1.42–9.79)	**0.032**
**Depth of myometrial invasion**				
<50%				
≥50%	3.64 (1.08–8.90)	0.151	2.82 (1.48–11.40)	0.202
**Cervical stroma** **involvement**				
No				
Yes	2.84 (0.75–12.71)	0.171	3.37 (0.98–11.11)	0.091
**Adnexal involvement**				
No				
Yes	2.56 (1.11–8.92)	0.097	2.02 (0.97–4.20)	0.061
**Serosal breach**				
No				
Yes	2.15 (1.01–10.54)	0.264	1.89 (1.18–13.60)	0.305
**Parametrial** **involvement**				
No				
Yes	2.88 (1.24–6.70)	0.064	3.20 (1.60–6.42)	0.081
**Pelvic lymph node involvement**				
No				
Yes	1.19 (1.02–1.71)	0.146	1.56 (0.77–3.16)	0.216
Unknown	2.20 (1.05–3.97)	0.106	1.08 (1.01–3.29)	0.103
**Para-aortic lymph node involvement**				
No				
Yes	3.52 (0.46–26.88)	0.221	3.82 (0.91–16.08)	0.067
Unknown	2.83 (0.89–8.97)	0.077	1.81 (0.66–5.00)	0.250
**LVSI**				
No				
Yes	1.94 (0.80–4.71)	0.142	1.83 (0.920–3.92)	0.118

HR, hazard ratio; S, serous endometrial carcinoma; E, endometrioid endometrial carcinoma; CC, clear-cell endometrial carcinoma; LVSI, lymphovascular space invasion; HTN, hypertension; DM, diabetes mellitus; COPD, chronic obstructive pulmonary disease. Statistically significant results are highlighted in bold (*p*-value < 0.05).

## Data Availability

Data are unavailable due to privacy restrictions.
